# Modeling Flow in an *In Vitro* Anatomical Cerebrovascular Model with Experimental Validation

**DOI:** 10.1101/2023.01.13.523948

**Published:** 2023-01-15

**Authors:** Saurabh Bhardwaj, Brent A. Craven, Jacob E. Sever, Francesco Costanzo, Scott D. Simon, Keefe B. Manning

**Affiliations:** 1 Department of Biomedical Engineering, Pennsylvania State University, University Park, PA, USA.; 2 Office of Science and Engineering Laboratories, Center for Devices and Radiological Health, U.S. Food and Drug Administration, Silver Spring, MD, USA; 3 Department of Engineering Science and Mechanics, Pennsylvania State University, University Park, PA, USA; 4 Department of Neurosurgery, Penn State Hershey Medical Center, Hershey, PA, USA.; 5 Department of Surgery, Penn State Hershey Medical Center, Hershey, PA, USA.

**Keywords:** Cerebrovascular model, Cerebral blood flow, Image based modeling, Acute ischemic stroke

## Abstract

Acute ischemic stroke (AIS) is a leading cause of mortality that occurs when a blood embolus becomes lodged in the cerebral vasculature and obstructs blood flow in the brain. The severity of AIS is determined by the location and how extensively emboli become lodged, which is dictated in large part by the cerebral flow and the dynamics of embolus migration that are difficult to measure *in vivo* in AIS patients. Computational fluid dynamics (CFD) can be used to predict the patient-specific hemodynamics and embolus migration and lodging in the cerebral vasculature to better understand the underlying mechanics of AIS. To be relied upon, however, the computational simulations must be verified and validated. In this study, a realistic *in vitro* experimental model and a corresponding computational model of the cerebral vasculature are established that can be used to investigate flow and embolus migration and lodging in the brain. First, the *in vitro* anatomical model is described and how the flow distribution in the model is tuned to match physiological measurements from the literature. Measurements of pressure and flow rate for both normal and stroke conditions were acquired and corresponding CFD simulations were performed and compared with the experiments to validate the flow predictions. Overall, the CFD simulations were in relatively close agreement with the experiments, to within ±7% of the mean experimental data with many of the CFD predictions within the uncertainty of the experimental measurement. This work provides an *in vitro* benchmark data set for flow in a realistic cerebrovascular model and is a first step towards validating an AIS computational model.

## 1. Introduction

Stroke is the second leading cause of mortality worldwide (over 6.5 million deaths/year) [1], with acute ischemic stroke (AIS) accounting for 87% of these [2]. AIS is a life-threatening medical condition that occurs when a blood embolus becomes lodged in the cerebral vasculature and obstructs blood flow in the brain. The available literature contains substantial research that has been performed to investigate cerebral blood flow [3-9]. The main focus of these studies was to investigate the regional cerebral blood flow, which forms the basis for identifying the cause of several diseases. However, the severity of AIS is determined by the location and how extensively emboli become lodged in the cerebral vasculature, but it is extremely challenging to measure the flow rate, pressure, and embolus migration in AIS patients. This level of information, however, is needed to elucidate the underlying causes and treatment of AIS.

The size and location of blood emboli in the cerebral vasculature have been the subject of several studies [10-12]. The middle cerebral artery (MCA) is the location where emboli most frequently lodge [13]. Measurements of the distribution of cerebral emboli within various arterial branches have been reported. A recent investigation using rats [14] showed that emboli shape and composition have a significant role in determining the severity of brain damage after they reach the cerebral vasculature. Using idealized Y-bifurcation geometries, Pollanen [15] and, more recently, Bushi et al. [16] conducted experiments on embolic particle migration. Beyond what volumetric flow patterns may imply, their research revealed a bias in the distribution of bigger particles into the broader branching arteries like the common carotid artery (CCA), internal carotid artery (ICA) and MCA. For their studies on embolus transport in the cerebral arteries, Chung et al. [10] used a more accurate anatomical model of the Circle of Willis and proposed a similar tendency of bigger particle sizes migrating toward the broader branching vessels. The distribution fraction of emboli across major arterial branches have been quantified in these experimental investigations corresponding to the associated volumetric flow patterns, but no information regarding fluid-clot-vessel interaction dynamics is elucidated.

Computational fluid dynamics (CFD) can be used to predict patient-specific hemodynamics and embolus migration and lodging in the cerebral vasculature to better understand the underlying mechanics of AIS and to improve recanalization methods. Patient-specific CFD has become widely used for simulating blood flow in the cardiovascular system [17]; examples include evaluating the hemodynamics of healthy and diseased blood vessels [18-19], assisting in the design and assessment of vascular medical devices [20-21], planning vascular surgeries, and predicting the results of interventions [22-23]. To be relied upon, however, the computational simulations must be verified and validated, which is difficult to achieve *in vivo*, particularly for embolus migration. Realistic *in vitro* models can be used to provide a rich data set for CFD validation. For example, researchers at the U.S. Food and Drug Administration (FDA) and collaborators have developed benchmark models of a simplified nozzle, a centrifugal blood pump, and a vascular model of the inferior vena cava and have acquired flow measurements that are widely used for CFD validation [24-29].

The objective of this study is to similarly establish an *in vitro* experimental model and a corresponding computational model of the cerebral vasculature that can be used to investigate flow and embolus migration and lodging in the brain. Levaraging a representative anatomical cerebral model, computational simulations are performed with particular focus on comparing with experimental pressure and flow rate measurements. In addition to normal physiological flow conditions, the effect of arterial embolus occlusion in the MCA is considered to emulate a stroke to investigate its influence on cerebral blood flow.

## 2. Methods

### 2.1. Cerebrovascular model

**Figure 1.**
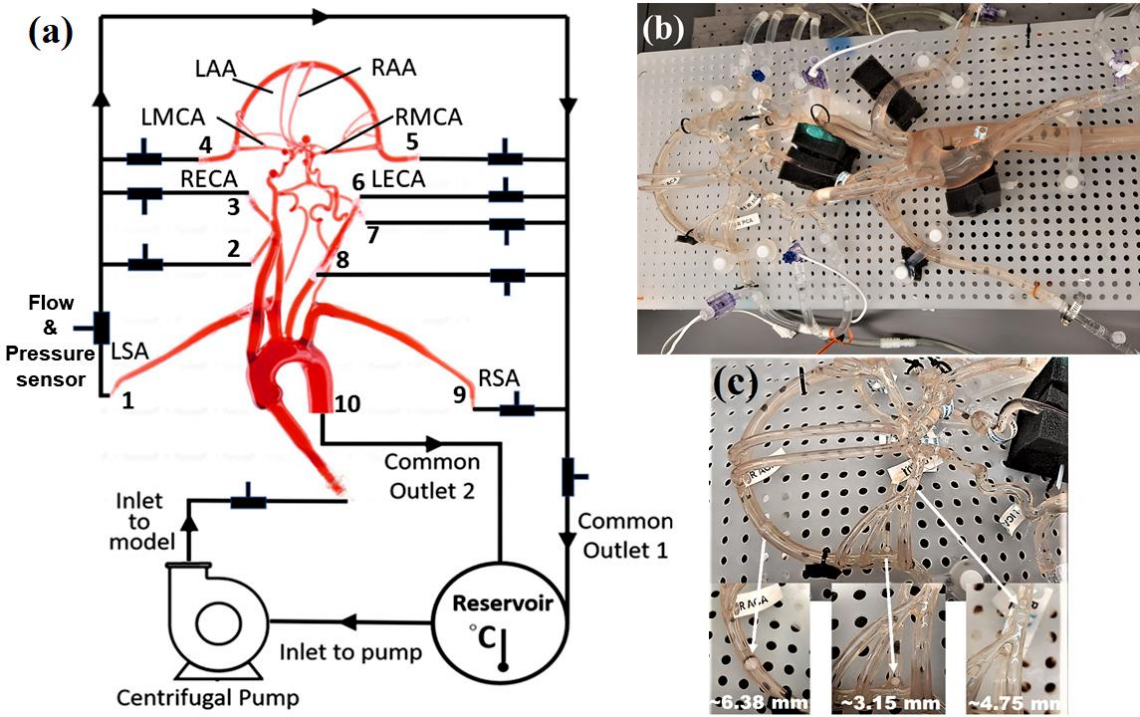
Circulatory flow loop (a) the experimental setup, (b) location of nylon spherical clots for mimicking a stroke condition and (c) schematic flow loop setup, to measure the steady flow and pressure data at various cerebrovascular outlets. Here, LSA and RSA are left and right subclavian arteries, LECA and RECA are the left and right external carotid arteries, LMCA and RMCA are the left and right middle cerebral arteries, and the LAA and RAA are left and right anterior arteries.

**Figure 2.**
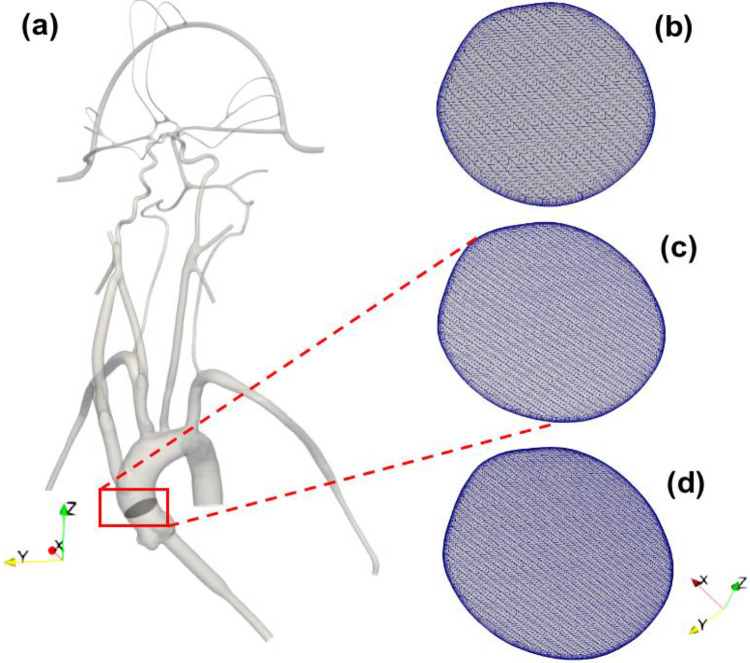
(a) 3D reconstructed computational model of the corresponding *in vitro* cerebrovascular model with a cut plane view in the aortic arch to show the mesh for the (b) 3 M, (c) 6.5 M and (d) 9 M cell meshes.

A representative anatomical model of the aorta and cerebral vasculature was used in this study (Fig. 1). The model was designed and fabricated by United Biologics (Irvine, CA, USA) based on patient medical image data from several sources (e.g., the NIH Visible Human Project, patient-specific CT data). The *in vitro* model (Fig. 1a) is made of silicone with an elasticit modulus of 3.1-3.4 N/mm^2^ that is representative of human arteries. The entire model includes the aorta, common carotid arteries, internal and external carotid arteries, axillary arteries, middle cerebral arteries, and anterior cerebral arteries. In addition, a corresponding computational model of the *in vitro* model was reconstructed from high-resolution 3D micro computed tomography (μ-CT) scans (Fig. 2a).

### 2.2. *In vitro* experiments

A closed-loop circulatory flow system was designed that included a centrifugal pump (Cole-Parmer, IL, USA), reservoir, flow meter and ultrasonic flow probe, and pressure transducers as shown in Figs. 1a & 1b. The flow loop was connected to the vascular model, which was placed in the supine orientation. The inlet and outlet flow rates and pressures were monitored using ultrasonic flow probes (Transonic Systems, Inc., Millis, MA) and pressure transducers (Merit Medical, South Jordan, UT), respectively. The pressure transducers were connected to an analog data acquisition module (DAQ, National Instruments, Texas, USA) and recorded using LabVIEW software (National Instruments, Texas, USA). The experiments were performed using a steady inlet flow rate of 5.17 ± 0.078 L/min, corresponding to a Reynolds number (*Re*) in the inlet tube of approximately 3890. The working fluid is a mixture of water (60% by weight) and glycerol (40% by weight) that is analogous to blood having a density and dynamic viscosity of 1.09 ± 0.03 g/mL and 3.98 ± 0.14 cP, respectively [26]. The inlet flow rate corresponds to a representative mean physiological cardiac output of an adult. An extended tube of 900 mm length was attached to the model inlet such that the flow entering the model inlet was fully developed. To study the effect of a stroke condition in cerebral arteries on the mean arterial pressure and flow rate, nylon spherical clots of three different sizes (i.e., diameters of 3.15mm, 4.75 mm, and 6.38 mm) were manually injected into the right MCA (RMCA) to completely block the vessel and the corresponding flow through outlet 4 depicted in Fig. 1c.

Three separate experiments for both conditions (i.e., normal and stroke) were performed to measure the flow rate and pressure at the inlet and various outlets in order to provide boundary conditions for the CFD simulation and for validation. Because the fluid was continuously pumped, we waited for 30 minutes to allow for the fluid temperature to reach a steady state (i.e., 22.2°C) before measuring the flow rate and pressure. Importantly, the viscosity of the fluid was tuned to account for the effect of this temperature rise, so as to obtain the desired value of 3.98 cP at the steady-state operating temperature. The regional distribution of the flow rate to the various arteries was tuned to match available literature data [30-31] by adjusting clamps downstream of the pressure and flow rate measurement locations. This yielded a regional flow distribution such that 73.2% of the flow passed through the descending aorta and 26.8% of the flow was distributed to the remaining arteries stemming from the aortic arch. The regional distribution of individual arteries provided in the literature is shown in Table 1. The flow rates to the various cerebral arteries were also tuned to match those reported in the literature [30-31].

### 2.3. CFD mesh

The computational geometry was discretized in CF-Mesh+ (version 3.5.1; Creative Fields, Croatia) using an unstructured hexahedral mesh with 5 wall-normal inflation layers at the walls to resolve large velocity gradients. Three different resolutions of meshes were generated yielding coarse, medium, and fine meshes having 3 million, 6.5 million, and 9 million computational cells, respectively (Figure 2). These generated meshes were used to perform a mesh refinement study to evaluate the sensitivity of the results to the mesh resolution. Using the *topoSet* utility in OpenFOAM, the mesh for the stroke condition was generated by removing the cells covering the section of the arteries that were blocked in the experiments by clots. After eliminating the cells at the clot injection sites, the newly created surfaces are converted to walls.

### 2.4. Governing equations & boundary conditions

Using the 3D reconstructed model, CFD simulations of the flow were performed by solving the Reynolds-averaged Navier-Stokes (RANS) equations using the open-source computational continuum mechanics library, OpenFOAM (version 2106). The gravitational force is not considered in the present simulations. The effects of turbulence were modeled using the two-equation eddy-viscosity k-ω shear stress transport (SST) turbulence model [32-33]. The k-ω SST model was chosen because of its reported good behavior in adverse pressure gradients and separated flows [34], and it has been shown to be capable of capturing laminar-to-turbulence transition in internal flows [35].

The CFD boundary conditions were specified to match the corresponding *in vitro* experiments. A constant, uniform inlet velocity was applied to the extended inlet to obtain fully developed flow entering the inlet to the ascending aorta at a flow rate of 5.17 L/min. A no-slip velocity boundary condition was applied on the walls, which were assumed to be rigid. The *pressureInletOutletVelocity* condition in OpenFOAM was applied for the velocity on all outlet boundaries, which uses a zero-gradient Neumann condition on boundary faces with outflow and an extrapolated Dirichlet condition on faces with reversed inflow. A zero-gradient pressure condition was applied at the inlet and fixed static pressure boundary conditions were prescribed on all outlets. The values of outlet pressure were specified by iteratively performing simulations to match the measured flow rate through each outlet in the experiments to within ±10%.

### 2.5. Numerical methods

Simulations were performed in OpenFOAM using second-order accurate discretization schemes. Preliminary steady simulations were performed by solving the steady RANS equations of motion using the semi-implicit method for pressure-linked equations (SIMPLE) solver, *simpleFoam*. The steady simulations did not converge owing to regions of significant unsteady flow in the ascending aorta and the aortic arch. Fully transient flow simulations were performed using the hybrid pressure-implicit with splitting of operators (PISO)-SIMPLE solver, *pimpleFoam*. An initial unsteady simulation was performed for 4 seconds of physical time, which was determined to be the time that was needed for the flow to overcome the initial startup transient conditions and to obtain a stationary, quasi-steady flow state. Simulations were then run for another 4 seconds at these quasi-steady conditions while time-averaging the flow solution. The simulations were performed on 40 compute cores of a high-performance computing (HPC) system at the Institute for Computational and Data Sciences at the Pennsylvania State University. Post-processing and visualization of the simulation results were performed in ParaView (Kitware, Inc., Clifton Park, NY).

## 3. Results

### 3.1. *In vitro* experiments

**Figure 3.**
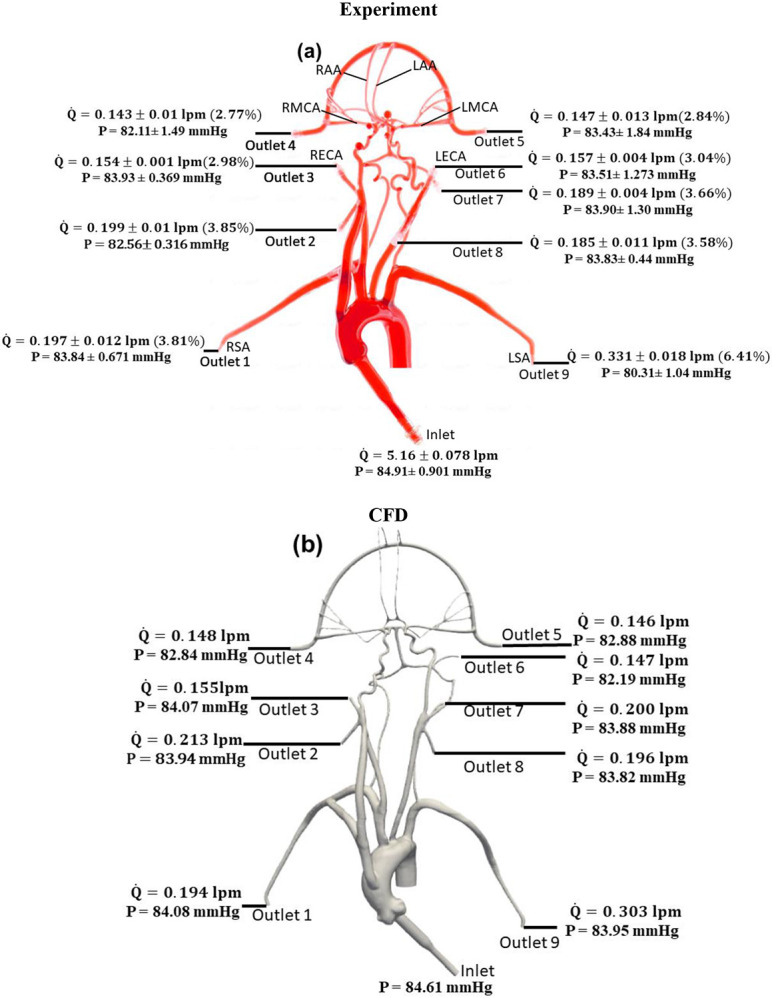
The mean volumetric flow rate and pressure values along with standard deviation at inlet and all arterial outlets from (a) *in vitro* experiments and (b) CFD simulation for the normal condition. The percentage values shown in parentheses indicate the fraction of total inlet flow in the individual arterial outlet.

The values of volumetric flow rate (L/min) and pressure (mmHg) measured at various arterial outlets are shown in Figure 3a. As mentioned in Section 2.2, the arteries stemming from the aortic arch, which includes the axillary, external, middle, anterior, and other arteries, receive 26.8% of the total flow rate entering the model and 73.2% of the fluid flows through the descending aorta. Out of the total flow rate, 10.23% travels through both axillary arteries, 6.02% through both external carotid arteries (ECA), and 5.62% through the middle and anterior cerebral arteries. This measured flow distribution matches the physiological values from the available literature [30-31]. However, it is important to mention that these distributions are based on the steady flow measurements, whereas the values reported in the literature are based on average values over a pulsatile cardiac cycle.

**Figure 4.**
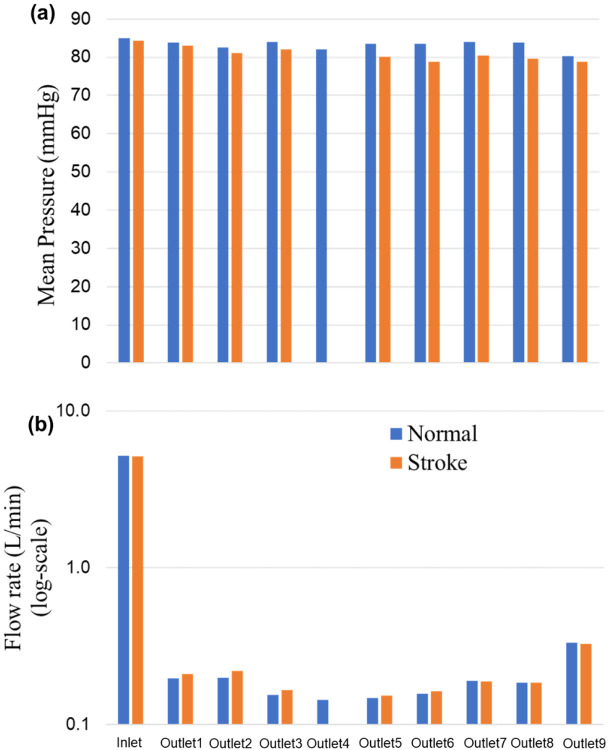
Comparison of mean pressure and flow rates (log-scale) at the inlet and various aterial outlets for the normal and stroke conditions. The values of pressure and flow rate at outlet 4 for the stroke condition are zero because there is no flow due to the blockage.

The changes in arterial pressure and volumetric flow rate were also measured for the stroke condition when there is no flow from outlet 4. The measurements showed that the pressure at all outlets decreased. As illustrated in Figs. 3 and 4, the flow rate through the right arterial outlets as well as outlets 5 and 6 increased to compensate for the flow that was obstructed due to the emulated stroke. A comparison of pressure and volumetric flow rate between normal and stroke conditions also showed that the mean pressure decreases at all outlets in the stroke condition compared to normal condition.

### 3.2. CFD flow simulation

Before commencing the CFD simulations using the present computational model, a rigorous mesh dependence study is conducted for three mesh sizes i.e., 3 mil (coarse), 6.5 mil (medium) and 9 mil (fine). The volumetric flow rate at arterial outlets corresponding to mean pressure is considered as control parameter for comparison in the normal conditions. The mean pressures are assigned as boundary condition at outlets and steady uniform volumetric flow rate is assigned as model inlet. The percentage change in the volumetric flow rate at outlet 1, outlet 3, outlet 5, outlet 6 and outlet 9 are calculated to be equal to ~1%, ~2%, ~0.28%, ~0.40% and ~0.29% between 6.5 & 9 mil meshes compared to ~3%, ~9%, ~2%, ~3% and ~1% between 3 & 6.5 mil meshes. The comparison of flow rate values show that the results for 6.5 mil and 9 mil meshes are in close agreement and therefore, eventually, 6.5M cells mesh is used for the final simulations for both normal as well as stroke conditions in the present study.

**Figure 5.**
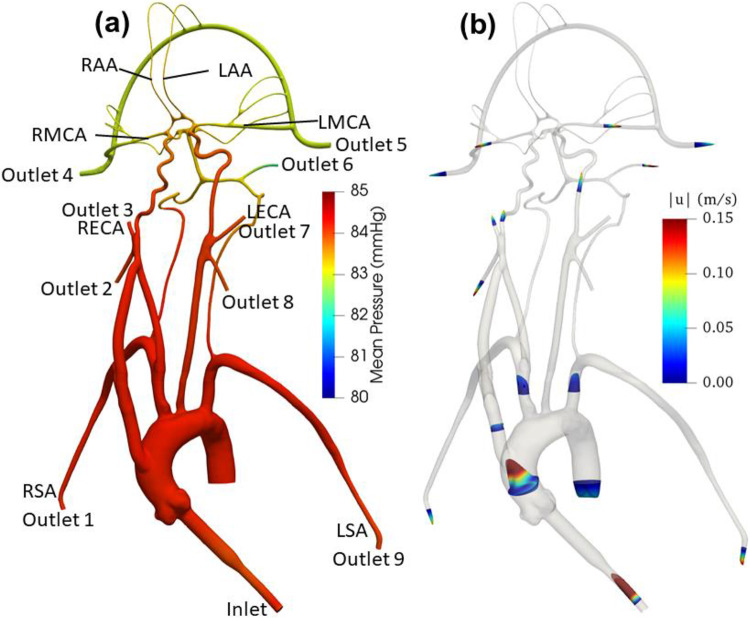
**(a)** The mean pressure contours and (b) profiles of mean velocity at various sections in the anatomical cerebral vascular model from CFD for normal condition.

Figure 5 illustrates the velocity contours in the cerebral vasculature model from CFD. The highest velocities are observed in the inlet. The flow speed in the aortic arch is also much higher compared to the more distal downstream arteries. From the CFD simulation, it is interesting to note that left common carotid has a higher velocity than the right common carotid. As the aortic root is attached with a smaller diameter inlet tube, there is some unsteadiness and recirculating flow in the downstream ascending aorta.

The mean pressure contours and velocity distribution in the model for the normal condition are shown in Figure 5, where higher pressure is observed in the aorta compared to the cerebral arteries. This is due to the fact that most of the pressure drop in the vascular model occurs in the smaller cerebral arteries

Quantitative values of volumetric flow rate and pressure at all outlets from the CFD simulation are shown in Figure 3b. The flow distribution in the axillary, external, and combined middle and anterior arteries are equal to 10.4%, 5.78% and 5.68%, respectively. Because outlet boundary conditions are assigned to match the experimental flow rate measurement through each artery outlet to within ±10%, the simulations are valiadted by comparing the resultant outlet pressures from CFD with the experimental pressure measurements. As summarized in Tables 2 and 3, all of the arterial outlet pressures are close to the experimental values to within ±7%, with many of the CFD outlet pressures within the uncertainty of the experimental measurement.

## 4. Discussion

The objective of this study is to establish a realistic *in vitro* experimental model and a corresponding computational model of the cerebral vasculature that can be used to investigate flow and embolus migration and lodging in the brain. Measurements of pressure and flow rate were acquired and corresponding CFD simulations were performed and compared with the experiments to validate the flow predictions. Overall, the CFD predictions were in relatively close agreement with the experiments, to within ±7% of the mean experimental pressure measurements with many of the CFD predictions within the uncertainty of the experimental measurement.

The *in vitro* model developed in this study represents a realistic anatomical model of the cerebovasculature and the upstream arteries that produce a realistic flow distribution. The measurements of the flow distribution are in close agreement with the range of clinical values reported in the literature [30-31]. Compared with other *in vitro* cerebrovascular models, the present model consists of the major cerebral vasculature and the aorta and aortic arch. In contrast, previous work [36-38] used models that did not incorporate all of the vasculature that is considered in the present work.

The present CFD simulations closely correspond with the *in vitro* experimental measurements of pressure in the various arterial outlets of the model. Interestingly, as found in [39-40], it is observed that the regional flow distribution in the cerebrovascular model was highly sensitive to the prescribed outlet pressure. In an exploratory sensitivity study, it is further observed that small variations in the prescribed outlet pressures within the range of the uncertainty of the experimental pressure measurements yielded extremely large variations in the regional flow distribution in the model. The flow pattern in the aortic arch in the current investigation correlates well with that reported in the computational study of Numata et al. [41]. The transient flow field found in the aortic arc in the present work has also been reported in other studies [42-45]. The previous work by Schollenberger et al. [46], which assessed flow and mean pressure in a image-based cerebral model including the aortic arch similar to the model used in the current investigation, found a similar mean pressure distribution in the aortic arch to cerebral arteries, as seen in the current study. Although the values of mean pressure differ somewhat from the current study, that is likely due to the differences in the inlet flow rate and the fully developed velocity profile used here.

Finally, there are several limitations of the present study that should be addressed in future work. First, only flow is measured and simulated in the model, and do not consider embolus migration and lodging, which is a topic of ongoing research. Additionally, steady flow is considered in this study, whereas the physiological flow in the aorta and cerebovasculature is pulsatile in nature. The objective of current research, however, is to provide a tiered validation data set for systematically validating computational simulations, the first step of which is to validate by comparing with steady flow. Also, the working fluid used in this study is Newtonian. The non-Newtonian rheology of blood is planned to be included in the future. Finally, the CFD simulations in this study assume that the walls of the vascular model are rigid, which is a reasonable assumption for steady flow through the model. Future work investigating pulsatile flow, however, should also consider the influence of vessel wall motion due to fluid-structure interaction and its influence on the flow and migration of emboli.

## Figures and Tables

**Table 1:** Distribution of cerebral volumetric flow rate from the literature. The flow rate values are reported as the mean ± standard deviation (SD).

Vessel	Flow rate (L/min)	References
Common carotid	8.5±0.9	[30]
	8.9	[31]
Internal carotid	5.3±1.0	[30]
Subclavian	8.1±0.8	[30]
Vertebral*	1.9±0.5	[30]
Axillary	6.2±0.9	[30]

* calculated difference between subclavian and axillary flow rates.

**Table 2:** Mean pressure (mmHg) obtained from *in vitro* experiments and CFD at various inlet and outlets for the normal condition. The experimental values are reported as mean ± standard deviation (SD) from three experiments.

Location	*In vitro* pressure (mmHg)	CFD pressure (mmHg)	% Difference
Inlet	84.91±0.90	84.61	−0.35
Outlet 1	83.84±0.67	84.08	0.28
Outlet 2	82.56±0.32	83.94	1.67
Outlet 3	83.93±0.37	84.07	0.17
Outlet 4	82.11±1.5	82.84	0.90
Outlet 5	83.43±1.8	82.88	−0.66
Outlet 6	83.51±1.27	82.19	−1.58
Outlet 7	83.90±1.30	83.88	−0.02
Outlet 8	83.83±0.44	83.82	−0.01
Outlet 9	80.31±1.04	83.95	4.53

**Table 3:** Mean pressure (mmHg) obtained from *in vitro* experiments and CFD at the inlet and outlets for the stroke condition. The experimental values are reported as mean ± standard deviation (SD) from three experiments.

Location	*In vitro* pressure (mmHg)	CFD pressure (mmHg)	% Difference
Inlet	84.25±0.79	84.64	0.46
Outlet 1	83.04±0.85	84.09	1.26
Outlet 2	81.01±0.72	83.84	3.49
Outlet 3	82.02±0.67	84.04	2.46
Outlet 4	-	-	-
Outlet 5	80.04±1.2	83.13	3.86
Outlet 6	78.83±1.7	82.19	4.26
Outlet 7	80.40±1.19	83.96	4.42
Outlet 8	79.55±0.96	83.89	5.45
Outlet 9	78.74±1.56	83.94	6.60

## Nomenclature

CCA: Common carotid artery
ICA: Internal carotid artery
ECA: External carotid artery
LECA: Left external carotid artery
RECA: Right external carotid artery
MCA: Middle cerebral artery
LMCA: Left middle cerebral artery
RMCA: Right middle cerebral artery
ACA: Anterior cerebral artery
LAA: Left anterior artery
RAA: Right anterior artery
LSA: Left subclavian artery
RSA: Right subclavian artery
